# A Comparison of Saliva and DNA Transfer and Persistence on Skin Immediately and 6 h Following Biting, Kissing, Licking and Sucking

**DOI:** 10.3390/genes17101229

**Published:** 2026-10-03

**Authors:** Cara Woollacott, Duncan Taylor, Roland A. H. van Oorschot, Mariya Goray

**Affiliations:** 1 College of Science and Engineering, Flinders University, Adelaide, SA 5042, Australia; cara.woollacott@flinders.edu.au (C.W.); duncan.taylor@sa.gov.au (D.T.); 2 Forensic Science South Australia, Adelaide, SA 5001, Australia; 3 Office of Chief Forensic Scientist, Victoria Police Forensic Services, Macleod, VIC 3085, Australia; 4 School of Agriculture, Biomedicine and Environment, La Trobe University, Bundoora, VIC 3086, Australia

**Keywords:** DNA transfer, DNA persistence, DNA-TPPR, activity-level evaluations, saliva, forensic biology

## Abstract

Background: The presence or absence of saliva and saliva-derived DNA may be relevant to a range of different crime types, including sexual assault and homicide offences, where allegations of saliva transfer to the skin of a victim or offender from actions such as biting, kissing, licking or sucking are relevant. The interpretation of skin samples following allegations of biting, kissing, licking or sucking requires an awareness of the prevalence, transfer and persistence of saliva, and its DNA composition, including self- and non-self DNA, with and without such activities occurring. The matter of persistence is especially important given that the examinations of suspects or victims do not often occur immediately after the offence. Methods: This study investigates the transfer of saliva onto human skin and its persistence following actions of biting, kissing, licking and sucking. Seven male–female couples were asked to shower and then bite, kiss, lick and suck separate defined areas of both of their partner’s arms. Following these different actions, one arm was sampled immediately, and the other arm was sampled 6 h later. Background (prior to shower) and control (post-shower but before deposition) samples were also obtained. For participants who wore long-sleeve clothing post-activity, the inner sleeves were also sampled along with the tested skin areas at 6 h sample collection. Presumptive saliva tests were carried out on all background, control, 6 h experimental and clothing samples collected, using the RSID-Saliva immunological test kits. All recovered samples (immediate, persistence, clothing, background and control) underwent DNA extraction and quantification, and all background, 6 h and clothing samples were STR-amplified, generating DNA profiles. Results: The results were considered with the questionnaire data provided by participants, to assess what factors may have influenced the presence or absence of any saliva, self (donor), partner or foreign DNA detected. Comparisons of the DNA quantities recovered from the immediate samples and 6 h samples were made. Furthermore, saliva detection rates of 6 h samples and DNA profile data were compared between all samples generated from the four different actions which resulted in saliva transfer to skin. Concussions: This research will assist forensic investigators when interpreting saliva DNA evidence recovered from the skin of victims or suspects after a prolonged period of time between the offence and collection.

## 1. Introduction

Saliva commonly transfers between individuals via direct, indirect and aerosol transfer during everyday interactions, sometimes unknowingly [1,2,3,4]. This is also a common occurrence within a range of different crime types, including both sexual assaults and homicides, where saliva is often transferred to the skin of a victim (either live or deceased) or an offender following a range of actions, including biting, kissing, licking and sucking [5,6,7]. Actions resulting in the transfer of detectable levels of saliva from one individual to the skin of another individual can provide valuable investigative information, particularly when evaluating evidence given activity-level propositions [8,9]. In 1999, Sweet and Shutter [10] exemplified the value of sampling bite marks in their case review of a homicide and sexual assault of a woman whose body was found after being submerged in water for 5.5 h. Forensic examiners sampled a bite mark impression left on her right breast, which revealed a mixed profile that ultimately identified the perpetrator in this case, highlighting the value of sampling skin areas [10].

However, the lack of available data to allow for proper interpretation of salivary DNA evidence can lead to acquittal, failure to commence or discontinued proceedings [11,12]. This was demonstrated by a Japanese case whereby a doctor was accused of sexual assault against a patient who alleged that he had licked her breasts, among other activities, while she was under general anesthesia during a procedure [11,13]. Despite positive α-amylase detection, and recovery of the defendant’s DNA from the complainant, he was ultimately found not guilty, partially due to questions surrounding how his salivary DNA was transferred to her skin, and if this was from criminal or non-criminal activity [11,13]. Evidence collected from a bite mark was also instrumental in the overturning of the wrongful conviction of John Kunco [14]. John Kunco was convicted in 1991 of rape and assault, largely based on the bite marks recovered from the victim’s body. However, DNA results from the bite mark, which were not available at the time of the trial, later excluded Kunco as the donor, which led to his release after 28 years in prison.

Current practices involve collecting swabs of skin areas to which suspected salivary DNA has transferred, from an alleged bite mark, or an area that has been spat on, kissed, sucked or licked during an offence [15,16]. However, the examination of a suspect or victim often does not occur immediately after the offence. Thus, data relating to the persistence of saliva on skin from a range of actions, and the resultant DNA profiles, including quantity and composition, can provide useful data for future interpretations of such evidence in a range of criminal offences [8]. Much of the existing research explores the persistence of saliva on skin and non-skin surfaces [5,17,18], providing optimized recovery methods [5,17] and comparing DNA extraction methods to improve profiles generated from saliva samples obtained from skin [18]. Other research has been conducted to investigate saliva-derived DNA from non-skin surfaces, including both synthetic man-made and natural substrates. Investigations of surface impact on saliva transfer and persistence focus on a range of porous (such as cotton and polyester sheets, pillowcases or clothing) and non-porous (such as plastic, glass and melamine) surfaces routinely encountered as evidentiary items [19,20,21,22,23,24,25,26]. It has been established that porous surfaces tend to absorb saliva, allowing for greater persistence, despite being exposed to environmental conditions which would normally reduce persistence (such as washing, subsequent contacts and other personal habits) and DNA recovery [24,27]. Further, research has shown that the use of a wet and dry double swabbing technique can effectively yield high quantities of DNA from saliva stains on porous surfaces, whereas the collection of saliva using only a single wet swab on non-porous surfaces is equally effective [27]. While valuable, applying information gained from transfer and persistence studies that focus on porous and non-porous substrates has limited applicability to saliva evidence collected from skin surfaces [8,24]. Hence, there is a need to generate data on the transfer and persistence of saliva and saliva-derived DNA to skin surfaces, including saliva detection rates and subsequent DNA results after different activities. Further, considering the activities which occurred between the initial transfer event and recovery of the sample can inform forensic casework where circumstances are comparable to those presented in this research [28,29].

The persistence of saliva deposited on skin surfaces after showering was investigated by Williams et al. [29], who successfully recovered salivary DNA using Y-STR analysis. Other research compared Chelex, organic and silica column-based extraction methods for the recovery of male saliva DNA that had been deposited onto the forearm of a female participant [18]. This study found that all methods were effective; however, silica column extraction methods yielded higher quantities of DNA with a success rate of 83% when carrying out amplification of 38 loci using the Ingenomics Y Profiler kit [18]. This study generated samples following biting, licking or spitting on the participants’ arms, highlighting the ability to generate useful DNA data from saliva transferred to skin [18]. Kenna et al. [5] investigated the recovery and persistence of saliva on human skin, by depositing 50 µL of neat saliva from male donors onto the legs of female participants. The authors then asked female participants to cover their legs with regular clothing and resume daily activities, without showering, before deposits were sampled using double swabbing and mini-tapes at 0 h, 24 h, 48 h, 72 h and 96 h post-deposition [5]. Their findings indicate that male DNA could be detected on the skin of female participants and generate a profile 96 h after initial deposition in 92% of instances. However, the concentration of DNA recovered 24 h post-initial deposition decreased by approximately 60% when compared to amounts recovered at 0 h [5]. These conclusions aligned with Sweet et al. [6], who observed a 40% loss of DNA recovered between 5 min and 24 h after saliva had been deposited onto the skin of cadavers. However, despite this decrease in DNA concentration, successful PCR amplification was achieved, generating usable profiles.

The present study aimed to simulate actions commonly occurring in a range of crime types which result in salivary DNA being transferred to a victim or offender, specifically from the actions of biting, kissing, licking and sucking of the skin. Volunteering male–female couples were asked to shower and then bite, kiss, lick and suck both of their partner’s arms. One of the arms was sampled immediately after deposition, and the other was sampled 6 h later. Participants returned to their regular activities during the 6 h period and were asked to complete a questionnaire addressing what they had done during that time. For participants who wore long sleeves, post-experiment and prior to the collection of 6 h samples, the inner sleeves were also sampled, in addition to the skin areas. Background DNA samples were obtained prior to showering, and control samples were collected after showering but before any saliva deposition occurred. Presumptive testing for saliva was completed on all background, control, 6 h experimental and clothing samples collected. The saliva test and DNA results were considered together with the questionnaire data. We hope that the generated data will assist forensic investigators when interpreting biological evidence recovered from a victim or suspect where saliva transfer to the skin occurs, relevant to a range of casework scenarios.

## 2. Materials and Method

### 2.1. Ethics

All participating individuals were over the age of 18 and provided informed consent prior to taking part in this research, in accordance with the Flinders University Human Ethics Committee (7745).

### 2.2. Experimental Design and Sample Collection

**Figure 1:**
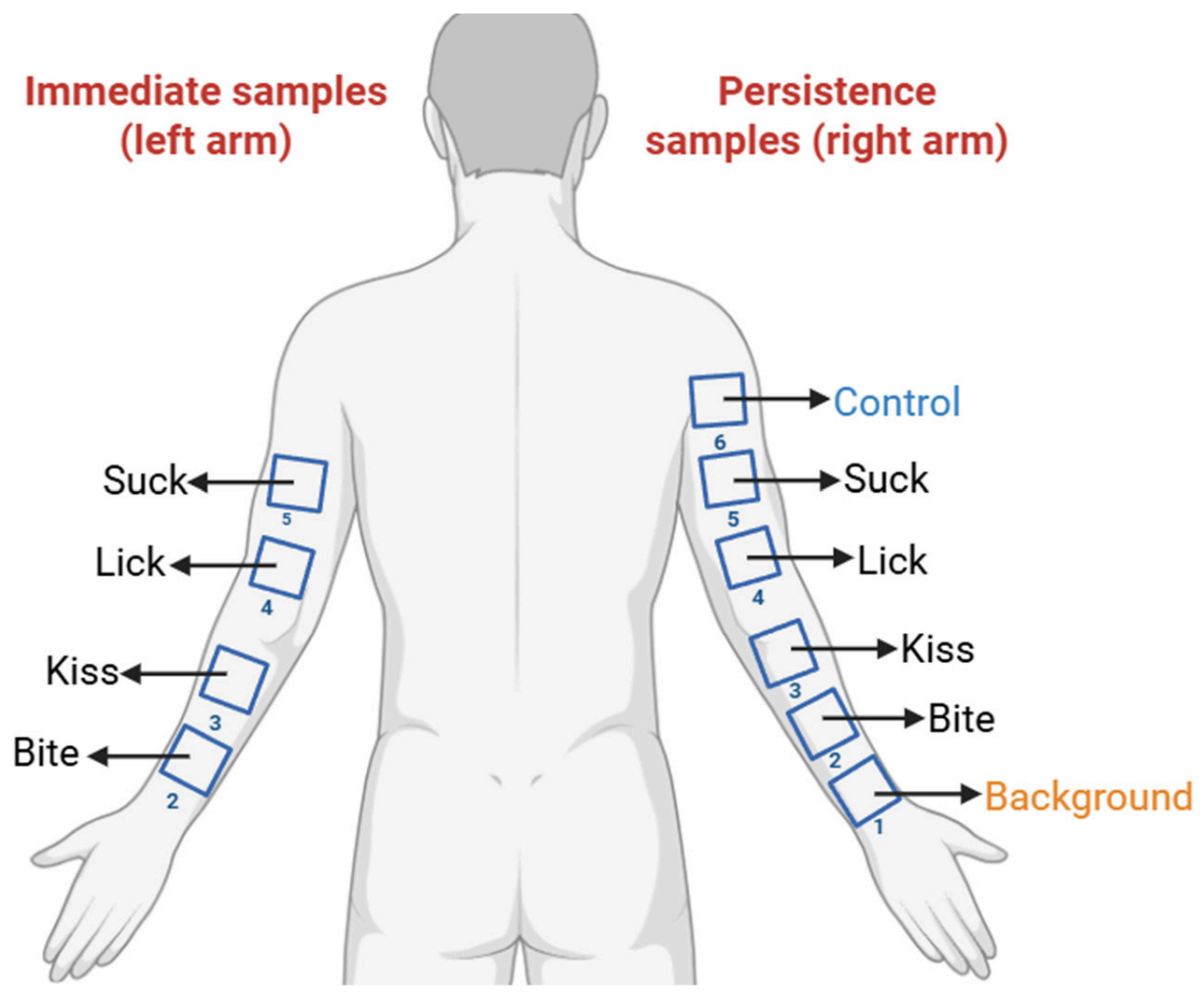
Diagram of the deposit squares marked and numbered along the outer arms of the donor participants prior to any activity (bite, kiss, lick and suck actions), whereby the elbow was avoided. Deposits to the left arms were sampled immediately after deposition and deposits to the right arm were sampled 6 h after initial deposition. The background sample (square 1 on right arm) was collected before showering, and the control sample (square 6 on right arm) was collected after showering but before any activity. Image created using BioRender.com.

Seven male–female couples volunteered to participate in this research, resulting in a total of 14 participants. Starting at the wrist and going up to their shoulders, six squares were marked and numbered from 1 to 6 along the participants’ outer right arms, using a non-water-soluble surgical marker and a 6 cm × 6 cm plastic stencil. A separate stencil was used to mark 4 squares along the outer left arm, which were numbered 2–5 (see Figure 1). All stencils were sprayed and wiped with 1% hypochlorite solution, deionised water, and 70% ethanol between each marking. The stencils were then UV-irradiated using the Bio-Link BLX crosslinker (Vilber Lourmat, Collégien (Marne-la-Vallée), France) for a total of 30 min, by laying them flat on one side, then turning the stencils over to the other side after 15 min. All samples obtained in this study were collected using a wet–dry double swabbing technique [30] with viscose swabs (Forensic Swab L, Sarstedt, Germany) and 2–3 drops of sterile water (Pfizer, Sydney, Australia). Swabbing was conducted by the researcher, whereby the swab was moved across each sampling area in a diagonal, vertical and horizontal zig-zag pattern in four alternating directions while continuously rotating the swab head for both wet and dry swabs. In this study, the “donor” is defined as the participant whose skin was sampled, and the “partner” is the individual who made the deposits. Both participants in the couple acted as “mock victim” and “mock offender”. When acting as the “mock victim”, the participant was kissed, bitten, licked and sucked by their partner termed the “mock offender”. The roles were then reversed. Samples collected from participants’ arms after they acted as a “mock victim” will be referred to as “donor samples”. When these same participants acted as “mock offenders” and kissed, licked, etc., the arm of their partner, they will be referred to as the “partner” in the samples collected from the donor’s arm.

First, a background sample (pre-wash sample) was collected from the square numbered 1 closest to the right-hand wrist of the participants. Participants were then asked to shower using warm water and soap for 5 min and dried off using a towel that had not been used since its last wash. A control sample (post-wash nonactivity sample) was then collected from the square labelled 6 closest to their right shoulder. Participants were then tasked to complete the following actions on their partner: bite square 2 (without leaving a mark or drawing blood), kiss square 3, lick square 4 and suck square 5, first on the left arm, and then on the right arm. Each of the actions lasted for 5 s. The squares on the left arm were sampled immediately after deposition actions. For the deposits on the right arm, participants were asked to wait 15 min for the saliva to dry before resuming their regular activities. Six hours later, the squares on the participant’s right arm were sampled to allow for some comparisons to the corresponding immediate samples recovered from the left arm. The inner sleeve of clothing, if worn, making direct contact with their right arm, was also sampled using wet and dry swabbing. Buccal swabs were also collected from all participating individuals, and researchers, for comparison. All samples collected were then returned to the lab and stored in paper envelopes at room temperature, prior to being processed.

### 2.3. Questionnaire Data

All donor participants were provided with a questionnaire to complete prior to showering in order to ascertain their demographic information and recent activities, with relevance to the background DNA samples collected. They were then provided with a second questionnaire to complete when post-activity samples were collected, to establish what activities during the 6 h period may have influenced the results. These questions covered whether clothing was worn, and if so, what type, and subsequent contacts to the sample areas (Supplementary Material S1). This data was assessed against the saliva and DNA results generated (Supplementary Material S2, sheet 1).

### 2.4. RSID™-Saliva Testing

All background, control, 6 h skin and clothing samples were tested for the presence of saliva to investigate the detection rate of saliva on skin and relevant clothing areas 6 h after each activity, using the RSID^TM^-Saliva immunochromatographic test kit (Independent Forensics, Lombard, IL, USA). This was conducted by cutting 1/8th of the wet and dry swabs combined into one sample that was then processed in accordance with the manufacturer’s instructions. Test kits were validated using neat saliva for the positive control and buffer only for the negative control, prior to any analysis of test samples collected. The results of control samples were as expected, revealing a single line along the control well for the negative control test, and two lines (one at the test and the other at the control well) for the positive control conducted. The same guidelines were used when interpreting RSID^TM^-Saliva test results for experimental samples, only reporting the final result 10 min after transferring 100 µL to the test cassette. A previously completed internal validation of the RSID^TM^-Saliva test indicates that positive reactions can be observed up to a 1/256 dilution for wet saliva samples and 1/64 dilution for dried saliva samples.

### 2.5. DNA Processing

All samples, consisting of a combined wet and dry swab, obtained during this study were extracted and quantified using DNA IQ™ (Promega^®^, Maddison, WI, USA) (final volume of 60 µL) and Quantifiler Trio™ on the QuantStudio™ 5 Real-time PCR system (ThermoFisher Scientific, Waltham, MA, USA), respectively, to measure total DNA amounts of each sample. Processing of all immediate and control samples took place at laboratory 2, with the remainder of samples being processed at laboratory 1 (Supplementary Material S4). The DNA IQ™ extraction protocols used were adapted from the Promega recommended isolation protocol from stains and buccal swabs [31]. The details of the extraction protocols used in each lab can be found in Supplementary Material S5, Extraction sheet, including differences in temperature and lysis buffer volume, introducing potential limitations. Amplification of the background, 6 h persistence and clothing samples was performed using PowerPlex 21^®^ system (Promega^®^, Maddison, WI, USA; 25 μL reactions, 30 cycles with a threshold of 175 RFU). The quantity of DNA amplified per sample was 0.5 ng or 15 μL if the concentration was ≤0.033 ng/μL. The profiles were generated using the 3500xL Genetic Analyzer (ThermoFisher Scientific, Waltham, MA, USA; 1.2 kv/24 s).

### 2.6. DNA Profile Interpretation and Data Analysis

Each DNA profile was read using Genemapper ID-X (v1.6) (ThermoFisher Scientific, Waltham, MA, USA) software and was assessed to determine the number of contributors based on the number of alleles and peak height information provided, taking into account stutters (e.g., double stutter, forward stutter, etc.) specific for the PowerPlex21 kit (Promega^®^, Maddison, WI, USA). All alleles and stutters were retained for deconvolution using STRmix™ statistical software (v2.9) (The New Zealand Institute for Public Health and Forensic Science (PHF Science), New Zealand, and Forensic Science South Australia (FSSA), Australia). Following deconvolution was the determination of mixture proportions and the generating of likelihood ratios (LRs). LRs of >1 were considered as inclusionary. We report the actual LR values as calculated, without inference or implication regarding their evidential weight. In all profiles, the major contributor was defined as the person contributing ≥70% of the total DNA from each sample, based on the STRmix™ mixture proportions. In samples where the donor (the participant whose arm or clothing was sampled) was not excluded as a contributor, these profiles underwent a second deconvolution where the donor was assumed.

The total amount of DNA in the sample was determined by multiplying the DNA concentration by the extract volume (60 µL). Total and male DNA quantities obtained from Quantifiler Trio™ quantification of all background, control, immediate, 6 h persistence and clothing samples were log10 transformed prior to statistical analysis. For amplified samples, mixture proportions generated by STRmix™ were used to establish self- (donor) and non-self DNA (partner and additional unknown) amounts (see Supplementary Material S2, sheet 3). For samples collected for each couple that had unknown contributors detected, mix-to-mix analysis using STRmix™ was performed to establish if any of the unknown donors were common to multiple samples with an LR threshold of 1000 to focus on comparisons providing strong support for a common donor. No theta correction was applied to mix-to-mix comparisons, as this is the standard calculation implemented in the software.

The primary outcome being investigated throughout the data analysis is to assess and compare DNA amounts transferred immediately after deposition when sampled at 0 h, and RSID test results in combination with DNA quantification and profile results from skin samples collected 6 h post-deposition. DNA transfer is the primary measured variable being investigated, whereby background and control samples are considered informative and clothing samples and questionnaire data are explanatory. Data normality was assessed via the Shapiro–Wilk test (Supplementary Material S3, sheet 2), including if parametric or non-parametric tests should be used. Statistical analyses were conducted using R studio (see Supplementary Material S4 for the complete R script), whereby the repeated measures taken from participants were accounted for within the statistical modelling. Where multiple pairwise comparisons are made, a correction factor (i.e., the Holm or Dunn’s method) was adopted and is reported in Supplementary Material S3, sheet 3. Each specific statistical test is included in Supplementary Material S3, sheet 2. All statistical analyses were conducted using R Studio (v 4.4.1). Further details of the methodology adopted into this research, including which methods were conducted at laboratory 1 and 2 involved in this study, have been expanded on in Supplementary Material S5. Experimental conditions and profile data per sample, as well as the details of the methodologies utilized, are provided in Supplementary Material S5 to ensure transparency and broader use of the data generated by this study, as per minimum standards for reporting DNA-TPPR data [32,33]. Furthermore, a crosswalk of the methods has now been added to Supplementary Material S5, sheet 1 for a clear summary of sample type, numbers and processing methods.

## 3. Results

### 3.1. General Findings

**Figure 2:**
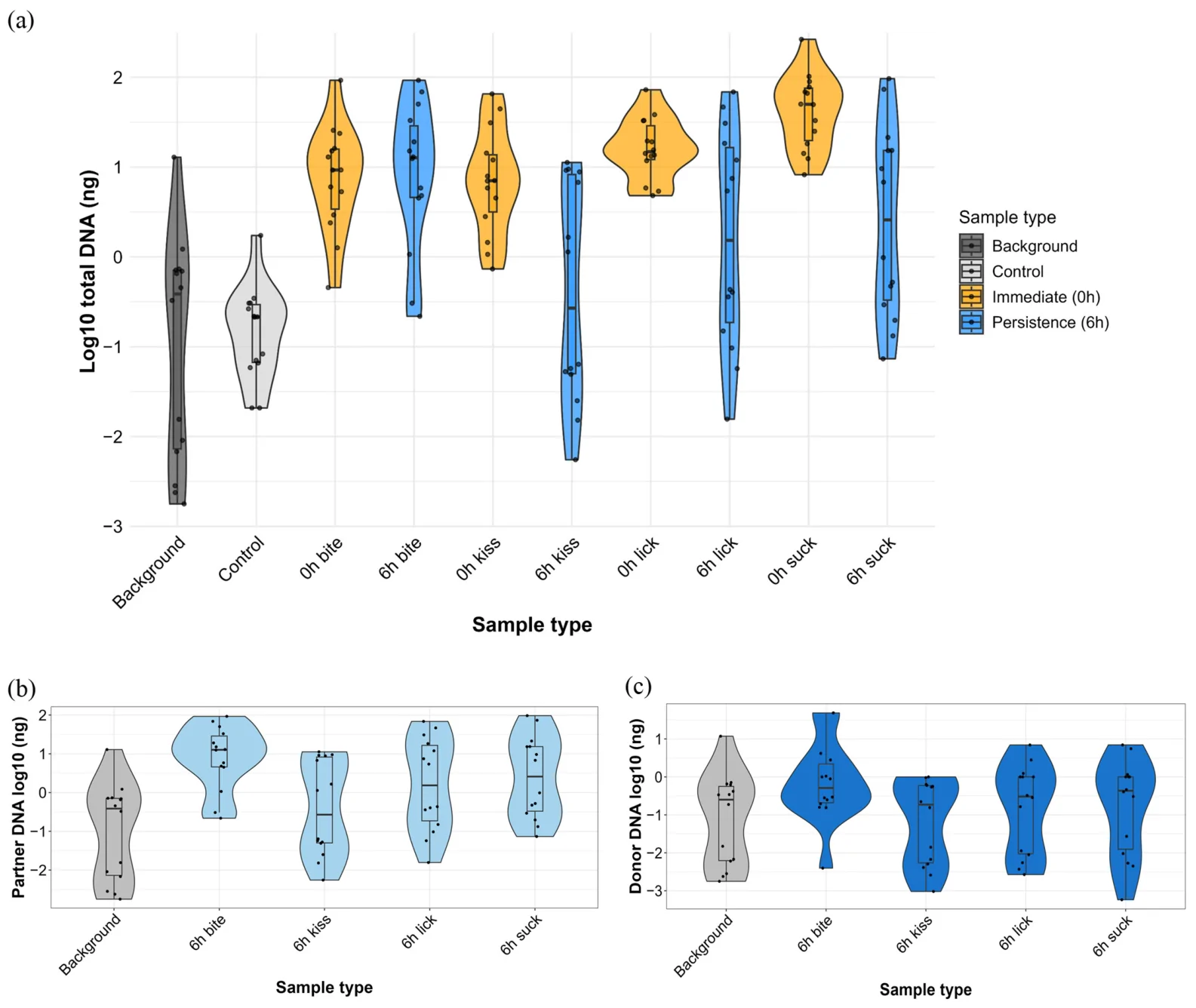
Median amounts of total (**a**) DNA (ng) detected in background, control, immediate and 6 h persistence skin samples; (**b**) DNA (ng) amounts attributed to partners in persistence samples; which is represented in light blue and (**c**) DNA amounts (ng) attributed to donors in persistence samples which is represented in dark blue. All partner and donor contributions were determined from STRmix mixture proportions. All samples were processed with Quantifiler Trio™ quantification kit.

A background sample was collected from the right wrist of each donor participant prior to showering, and a control sample was collected from the right shoulder of each donor participant after showering but prior to any saliva deposition. Therefore, a total of 14 background and 14 control samples were collected during this study and underwent DNA extraction and quantification. For background samples, the total amount of DNA ranged from 0.002 ng to 12.88 ng, with an average of 1.27 ng of DNA (SD 3.37 ng; median amount of 0.39 ng; Figure 2). Comparatively, the total amount of DNA recovered from control samples ranged from 0.02 ng to 1.73 ng, with an average of 0.28 ng (SD 0.43 ng; median value of 0.21 ng).

A total of 112 activity samples were collected from a bite, kiss, lick or suck action made on the skin of a participant during this research. Of the 56 immediate samples analyzed, the average amount of total DNA detected was 28.72 ng (SD of 41.33), ranging from 0.46 ng to 264.36 ng. Comparatively, the average amount of DNA detected across the 56 activity samples collected 6 h post-deposition (excluding clothing) was approximately 14.30 ng (SD of 23.75), ranging from approximately 0.006 ng to 96.51 ng of DNA. The median amount of total DNA detected on all immediate samples collected was approximately 14.19 ng, compared to a median value of 5.12 ng of DNA on the 6 h persistence samples.

The 14 control samples were processed up to and including quantification, measuring total and male DNA. Figure 2a offers a comparison of total DNA quantities in background, control, immediate and persistence skin samples, while b and c offer a comparison of partner and donor DNA amounts detected in skin samples that were STR-profiled. All DNA profiles from background, 6 h persistence samples and clothing samples were analyzed via STRmix to determine the inclusion of the donor and partner in profiles with two or more contributors. When donors were not excluded from the mixtures (LR > 1), they were then assumed for the second deconvolution. The majority of the inclusionary LRs (80%) were >1 million, with the remaining ranging from 1.1 to 1 million.

**Figure 3:**
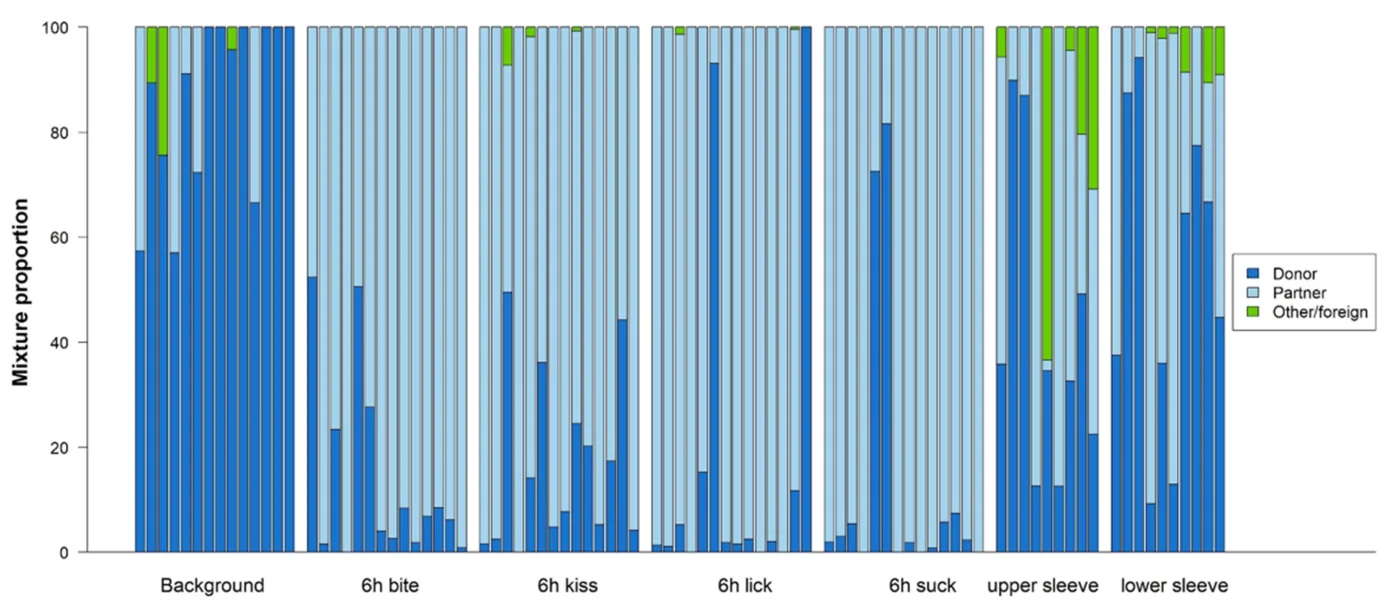
Mixture proportions of donors and partners and others (including unknowns) observed on all background, bite, kiss, lick and suck samples collected 6 h post-deposition, including single-source (15/70) and mixed profiles (55/70).

The background samples all generated a profile with the number of contributors ranging from 1 to 2 (see Supplementary Material S2, sheet 3). Six background DNA samples, which revealed only one contributor, generated an LR, supporting the inclusion of the donor (see Supplementary Material S2, sheet 3). Eight background samples revealed two-person mixtures. For all background samples that generated mixed profiles, donors were included, and thus, an average mixture proportion of 70% of the total DNA was generated, with the donor detected in all mixed samples. However, non-donor proportions attributed to partners represented an average of 40% of total DNA where there was more than one contributor detected. Only a single sample had non-donor DNA present in major proportions, whereby the donor was 28% of total DNA detected. Of the eight mixtures, five generated an inclusionary LR along with the donor. The average LR for partners ranged from 8.47 × 10^4^ to 5.22 × 10^24^. One background sample generated an LR of 740 (representing 4% of total DNA based on mixture proportions), supporting the inclusion of a friend (who provided a reference profile) in addition to the donor, while two samples had donor and unknown DNA, whereby unknown DNA represented 11% and 24% of the total DNA based on mixture proportions. Figure 3 displays a visual comparison of mixture proportions observed across all samples that were profiled.

A significant difference between the total DNA amounts was detected when comparing all sample types (control, background, immediate, bite, kiss, lick and suck, 6 h bite, kiss, lick and suck, and clothing samples) (*p*-value = 5.04 × 10^−11^, epsilon-squared d = 0.43). When comparing total amounts of DNA detected from all immediate activity samples to all 6 h persistence samples, significantly greater amounts of total DNA were observed on all bite, kiss, lick and suck samples recovered immediately after deposition compared to those recovered 6 h post-deposition (*p*-value = 9.07 × 10^−8^, d = −1.18, Figure 2).

**Figure 4:**
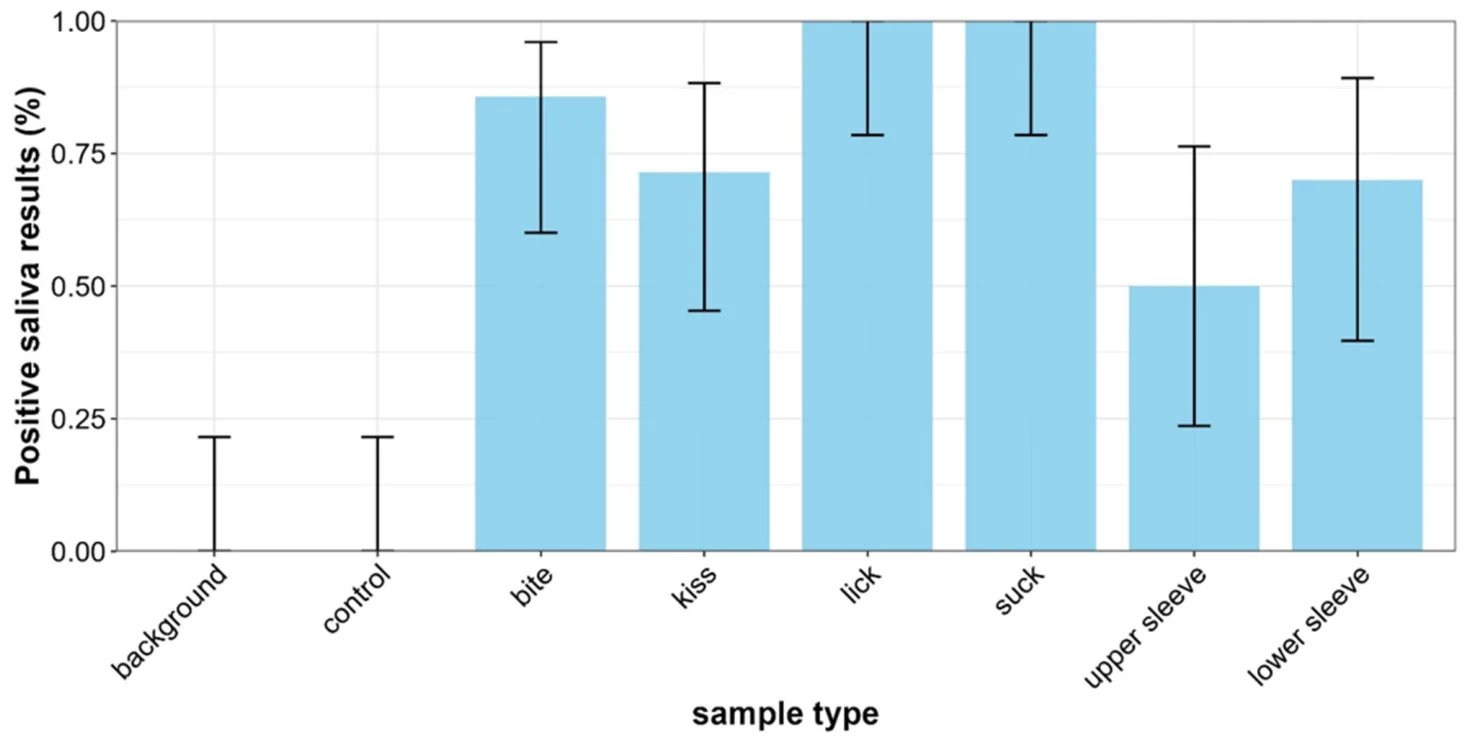
Proportions of positive RSID^TM^-Saliva results observed across background (0/14) samples, control (0/14) samples, and all 6 h samples (bite (12/14), kiss (10/14), lick (14/14) and suck (14/14), upper sleeve (5/10) and lower sleeve (7/10) samples) tested for the presence or absence of saliva. The error bars indicate 95% Wilson binomial confidence intervals.

All background, control, 6 h persistence and clothing samples underwent presumptive testing for saliva, using the RSID™-Saliva immunological test kit to investigate the detection rate of saliva 6 h after each activity. All background and control samples produced a negative saliva result. This differed greatly from the 6 h persistence samples, of which 89% generated a positive test result. Further, out of 20 clothing samples tested for the presence of saliva, 60% generated a positive test result, 5 from the upper sleeves and 7 from the lower sleeves of participants’ clothing. The frequencies of positive and negative saliva results per sample type are summarized in Figure 4 (Supplementary Material S2, sheet 2).

Of all the samples which underwent DNA amplification (*n* = 90; background (*n* = 14), 6 h (*n* = 56) and clothing (*n* = 20) samples), complete profiles were generated from 99% of the samples (89/90 samples). A single clothing sample did not generate a profile and therefore was excluded from subsequent analyses. The number of contributors ranged from one to four. For the 16 samples where 3–4 contributors were determined, 5 profiles were generated from participants’ skin (licking and kissing actions), with the remaining profiles all generated from clothing samples (*n* = 11). The highest number of contributors observed was a four-person mixture (single sample) obtained from a clothing sample.

### 3.2. Bite Samples

Immediate bite samples revealed an average of 15.93 ng of DNA (SD 23.43 ng; median of 9.28 ng of DNA), ranging from approximately 0.46 ng to 92.52 ng of DNA. Comparatively, 6 h after the biting action, there was an average of 22.92 ng (SD 28.48 ng; median value of 12.69 ng) of DNA detected, ranging from approximately 0.22 ng to 92.52 ng of DNA. Significantly more DNA was detected on immediate and 6 h bite samples than on the control (*p*-value = 1.15 × 10^−7^, d = 3.1, and *p*-value = 2.01 × 10^−6^, d = 1.6, respectively) and background samples (*p*-value = 1.43 × 10^−4^, d = 1.13, and 2.34 × 10^−4^, d = 1.17). However, while on average more DNA was detected in immediate samples compared to 6 h samples, these differences were not significant (*p*-value = 0.92, d = 0.02).

The bite samples collected after 6 h were processed and all generated complete STR profiles. Out of 14 samples, all but 1 generated a two-person mixture. Donors and partners were not excluded from all mixtures. The partner was not excluded and hence considered the only contributor of the single-source profile. The partner LRs ranged from 6.9 × 10^22^ to 2.8 × 10^27^ for mixed DNA profiles. On average, the partners represented 85% of the total DNA. Comparatively, for the donors, the average mixture proportion was 15%.

The difference between donor and partner mixture proportions in the bite samples recovered after 6 h can be visualized in Figure 3. The average amount of self-DNA detected from these samples was approximately 4.56 ng of DNA (SD 12.78 ng; median of 0.28 ng), ranging from 0.004 to 48.45 ng of DNA. In contrast, the average amount of non-self DNA, which could be attributed to partners in all 6 h bite samples, was 18.69 ng of DNA (SD 21.39 ng; median of 11.16 ng of DNA), ranging from 0.15 ng to 68.13 ng.

### 3.3. Kiss Samples

The 14 kiss samples which were recovered immediately after deposition had an average of 14.69 ng of DNA (SD 19.2 ng; median of 7.04 ng), ranging from approximately 0.73 ng to 65.28 ng of DNA. The 6 h samples had an average of 3.47 ng of DNA (SD 4.47 ng; median of 0.60 ng), ranging from 0.006 to 11.25 ng of DNA. Significantly more DNA was recovered from immediate kiss samples when compared to the control samples (*p*-value = 2.87 × 10^−6^, d = 2.43), unlike comparisons between control and 6 h kiss samples, which revealed no significant difference in DNA amounts (*p*-value = 0.43, d = 0.33). When comparing the immediate and 6 h samples to the amounts of DNA detected in the background samples, significantly more DNA was detected in immediate samples (*p*-value = 2.33 × 10^−4^, d = 1.58) and 6 h persistence samples (*p*-value = 0.02, d = 0.85). Statistical analysis also established a significant difference between the total DNA amounts detected between immediate and 6 h kiss samples (*p*-value = 2.83 × 10^−3^, d = −1.19), with lower quantities of DNA detected after 6 h.

The 6 h samples also underwent DNA profiling, all generating results with 1–3 contributors (average of 2). The donor could not be excluded from 13 of these profiles while partners were not excluded from all 14 samples, including one sample that produced a single-source profile. For the partners, LRs ranging between 8.01 × 10^3^ and 1.68 × 10^27^ were generated. For mixed samples, the average mixture proportion for partners was 84%, compared to 16% for donors. For the three samples where a third, unknown contributor was detected, these donors represented on average 3% of the total DNA.

Figure 3 displays a direct comparison of the donor and partner mixture proportions generated from 6 h kiss samples. There was an average of 0.24 ng of self-DNA detected (SD 0.32 ng), with a median of 0.01 ng, ranging from 0.003 ng to 0.96 ng of DNA. Greater amounts of DNA were attributed to non-self DNA partner contributions, with an average of 3.24 ng (SD 4.38 ng; ranging from 0.004 ng to 10.7 ng; median of 0.27 ng). Comparatively, DNA identified to be from an unknown source ranged from 4.35 × 10^−4^ ng to 0.12 ng (average of 0.07 ng; median of 0.08 ng).

### 3.4. Lick Samples

For the 14 immediate lick samples, an average of 21.41 ng of DNA (SD 18.09 ng) was recovered, with a median of 14.85 ng, ranging from 4.81 ng to 72.54 ng of DNA. The lick samples recovered after 6 h had an average of 13.62 ng of DNA with a median of 2.92 ng, ranging from 0.02 ng to 68.59 ng of DNA (SD 21.10 ng). Significantly more DNA was recovered after the licking action (both the immediate and 6 h) when compared to control samples (*p*-values = 1.42 × 10^−8^, d = 3.74; and *p*-value = 0.02, d = 0.85, respectively). This was also the case with background and immediate samples (*p*-value = 1.07 × 10^−4^, d = 1.76) and background and 6 h samples (*p*-value = 1.49 × 10^−4^, d = 1.68). Furthermore, significantly greater amounts of total DNA were detected in immediate samples when compared to those collected 6 h later (*p*-value = 0.02, d = −0.87).

All 6 h samples generated DNA profiles with the number of contributors ranging from one to three (average of 1.9). The donors could not be excluded from 11 of the 14 profiles (79%). In the remaining three samples, the partner was detected as a single contributor. The partner was not excluded from 13 of the 14 profiles (93%), including 3 single-source profiles. For the partners, LR ranged between 5.31 × 10^17^ and 1.76 × 10^26^. Of the two samples that generated three-person mixtures, one profile was a mixture inclusionary of the donor, partner and an unknown contributor (representing approximately 1% of total DNA). The other profile was a mixture inclusionary of the donor, partner and a co-habitant; however, this co-habitant provided very low inclusionary support (LR of 1.2 and mixture proportions of 0.44%).

The average mixture proportion for donors in all 6 h samples, excluding single-source profiles, was 14% compared to an average of 88% for partners (excluding three single-source profiles) (Figure 3). Of the 11 samples where donors were detected, an average of 1.11 ng (SD 1.91 ng) was attributed to self-DNA, ranging from 0.004 ng to 6.95 ng (median value of 0.29 ng). This was less than the quantities of partner non-self DNA, which generated an average of 13.72 ng of DNA (SD 21.05 ng) and a median value of 0.47 ng, ranging from 0.002 ng to 67.36 ng. The amount attributed to the unknown donor was 0.0004 ng of DNA.

### 3.5. Suck Samples

An average of 62.86 ng of total DNA (SD 65.45 ng) and a median of 49.94 ng—ranging from approximately 8.22 ng to 264.36 ng—was recovered from samples collected immediately after the sucking action. This was higher than the DNA amounts recovered 6 h post-deposition, where on average 17.21 ng were recovered (SD 29.89 ng), ranging from 0.07 ng to 96.51 ng, with a median of 3.88 ng. Significantly higher amounts of DNA were recovered from immediate and 6 h samples compared to control samples (*p*-value = 2.97 × 10^−10^, d = 5.01; and *p*-value = 3.42 × 10^−3^, d = 1.14, respectively). Further, significantly more DNA was also recovered from immediate and 6 h samples when compared to background samples (*p*-value = 1.00 × 10^−5^, d = 2.18; and *p*-value = 1.61 × 10^−4^, d = 1.65). Additional statistical testing confirms that significantly more DNA was recovered from immediate samples than 6 h samples (*p*-value = 3.02 × 10^−4^, d = −0.12).

The 6 h samples were profiled, and all generated DNA results with number of contributors ranging from one to two (average of 1.7). Partners were not excluded from all 14 samples including four single-source profiles, generating LRs ranging from 9.7 × 10^24^ to 2.48 × 10^27^, from which the donor was excluded. For the mixtures, both donors and partners were not excluded, with the donors contributing an average of 18% and partners contributing an average of 82% of total DNA (Figure 4). The partner LR’s ranged from 3.98 × 10^20^ to 1.48 × 10^27^ (Supplementary Material S2, sheet 3). On average, 1.45 ng of total DNA was attributed to self (SD 2.58 ng; median of 0.16 ng, ranging from 0.0006 ng to 6.98 ng) and 16.17 ng of DNA was attributed to partner non-self DNA (SD 30.25 ng; median of 1.12 ng; ranging from 0.07 ng to 96.51 ng).

### 3.6. Clothing and Questionnaire Results

Eleven participants reported wearing clothing, covering at least one deposit during the 6 h period before sampling. A total of 20 clothing samples (one of the eleven individuals did not provide their clothing for sampling) were taken from these participants: 10 from the inner layer of their upper sleeves (covering the lick and suck deposits) and 10 from the inner layer of the lower sleeves (covering the bite and kiss deposits). Clothing samples were obtained as a means of explaining any possible loss or gain of DNA to the skin areas sampled and should not be interpreted as outcomes of secondary transfer from skin to clothing due to previous wear of these garments and lack of background samples. The results reveal that significantly more DNA was recovered from skin samples collected 6 h post-activities compared to clothing samples (*p*-value = 0.04, d = 0.04, Supplementary Material S3, sheet 2). The total amount (*p*-value = 3.02 × 10^−1^, d = 1.06) and partner DNA (*p*-value = 0.32, d = 0.97) detected on the skin of participants was not found to be significantly different than DNA amounts detected on clothing. It was established that median amounts of partner DNA recovered on participants’ 6 h skin samples and the same participants’ clothing samples had greater amounts of partner DNA (*p*-value = 1.91 × 10^−2^, d = −0.85). Tables S1–S3, and Figures S1 and S2 in Supplementary Material S2, sheet 5, offer a direct comparison of the partner DNA amounts detected per clothing sample and their corresponding skin samples.

Furthermore, of all upper and lower sleeve samples tested for the presence of saliva, 60% generated a positive saliva test result (5/10 from the upper sleeve and 7/10 from the lower sleeve) (Figure 4). Statistical analysis found a significant relationship between the amount of partner DNA and a positive or negative saliva test result on clothing samples, finding that samples with a positive saliva result had a greater quantity of partner DNA detected (*p*-value = 2.15 × 10^−2^, d = −0.54, Supplementary Material S3, sheet 2).

Profiles were generated from 19 of the clothing samples, with the number of contributors ranging from two to four (average of 2.6). One sample did not produce a profile and was excluded from further analysis. Donors and partners were detected in all 19 samples representing the majority of DNA in all clothing samples. Of the 11 samples that contained unknown DNA (including a single four-person mixture with two unknown contributors), foreign DNA mixture proportions represented an average of 8% of their respective profiles. On average, the donors contributed 48% and partners contributed an average 46% of total DNA, with partner LRs ranging from 3.02 × 10^3^ to 1.15 × 10^27^. Mixture-to-mixture analysis identified a single possible common unknown donor detected on the upper and lower sleeves of the same garment (participant M; LR of 5450). No other common donors were detected between the clothing areas and corresponding skin samples (including background samples) taken from donor’s arms above the reporting threshold.

**Figure 5:**
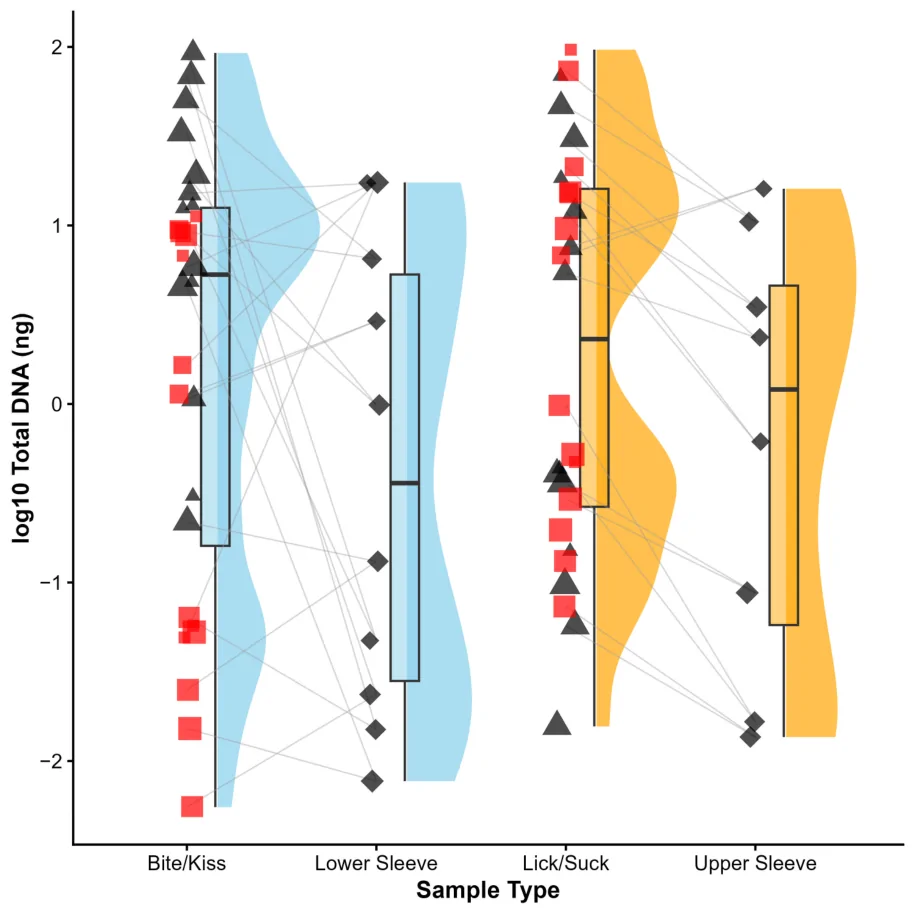
Distribution and frequency of total DNA amounts log10(ng) detected on all 6 h activity samples compared to the clothing samples. The bite/kiss persistence samples were paired with the lower sleeve, and the lick/suck samples were paired with the upper sleeve based on their positioning on the participants’ arms. Samples from the same experiment have been connected with a grey line. The size of the points reflects the amount of time clothing was worn (ranging from the smallest point, being 0 h, to the largest point, being 6 h). In the bite/kiss column, the bite samples are represented by red squares, and the kiss samples are represented by black triangles. In the lick/suck column, the lick samples are represented by red squares, and the suck samples are represented by black triangles. All other points (lower and upper sleeves) are represented by black diamonds. The blue raincloud represents the DNA distributions between bite/kiss deposits and the lower sleeve samples, and the orange raincloud represents the DNA distributions between lick/suck deposits and the upper sleeve samples.

The total DNA amounts ranged from 0.01 ng to 17.41 ng of DNA (average of 4.13 ng; SD 6.31 ng; median of 0.62 ng), excluding the one sample that did not generate a profile and produced a negative quantification result. Figure 5 provides a visual comparison of the total DNA amounts detected on clothing samples, compared to activity samples recovered from those who reported wearing clothing during the 6 h prior to sample collection.

Using the questionnaire data, a series of statistical analyses were conducted to establish what may have influenced DNA amounts recovered from the 6 h deposits, besides wearing clothing. To assess if contacts with the deposits had influence on the results, participants were asked if they had directly gripped, rubbed, scratched, and/or lightly touched any of the test areas numbered 2–5. Across all 6 h bite, kiss, lick and suck deposits, participants reported gripping (*n* = 4), rubbing (*n* = 16), scratching (*n* = 8), touching (*n* = 4), scratching and rubbing (*n* = 12) and gripping and rubbing (*n* = 2) the areas tested. However, no statistical significance was observed when assessing an association between the amounts of total and partner DNA detected in each sample and the reported combination of contacts made to each area (each individual *p*-value is reported in Supplementary Material S3, sheet 2).

For the participants that wore clothing, each reported how long, if at all, they had each deposit covered, and from which time point it was either exposed or covered (e.g., from hour 4–6 during the 6 h period between deposition and recovery). One participant (participant D) reported covering the deposits from hours 2–6. All other participants wore clothing from hour 0 onwards, for either 2.5 h (*n* = 1), 3 h (*n* = 2), 4.5 h (*n* = 1), 5 h (*n* = 3), or the entire 6 h (*n* = 2), covering all deposits. Notably, the participant that did not provide their clothing to be sampled (participant K) had the suck (square 5) deposit covered for the entire 6 h, and the others left exposed. Statistical analysis found a significant association between the duration each deposit was covered and the amount of partner DNA detected on the skin deposits after 6 h, finding a decrease in DNA amount as wear time increased (*p*-value = 3.30 × 10^−4^, d = 0.31). When looking at partner DNA amounts on clothing samples, the duration that the deposits were covered did not significantly alter the amounts of partner DNA detected; however, a general decrease in the amount was noted (*p*-value = 0.061, d = 0.61, Supplementary Material S2, sheet 5, Figures S1 and S2). Further, a post hoc analysis can be seen in Tables S4 and S5 in Supplementary Material S3, sheet 3. Notably, the sample size of all categorical or descriptive variables gained from questionnaire data (e.g., age range of participants and clothing type/material), and the generalization of sample data collected from couples participating in this study, are limited and only representative of an exploratory sample group, not representative of a population.

### 3.7. Activity-Level Evaluation Example

The data generated in this study can be used for activity-level evaluations in forensic investigations where allegations of biting, sucking, kissing or licking during an offence have been made. Here, we present an example of how this data might be used in an evaluation considering the following scenario:

The female complainant is in an intimate relationship with the male defendant. They are cohabiting roommates. The complainant has alleged to police that the defendant forcefully bit her on the arm during an assault which occurred 6 h prior; however, the defendant denies these allegations. Police recovered swabs of the complainant’s arm from the area she alleged was bitten, and these were sent to the forensic department for analysis.

The data presented here has been organized according to the method presented by Taylor et al. [34]. The activity-level propositions are:

**H1.**  *The defendant bit the arm of the complainant during an assault.*

**H2.**  *The defendant did not bite the complainant during an assault.*

Background:

The complainant and defendant cohabitate.

The complainant and defendant were not intimate in the past day.

Assumptions:

The complainant and defendant were together within the 6 h between the alleged assault and the complainant being processed.

**Figure 6:**
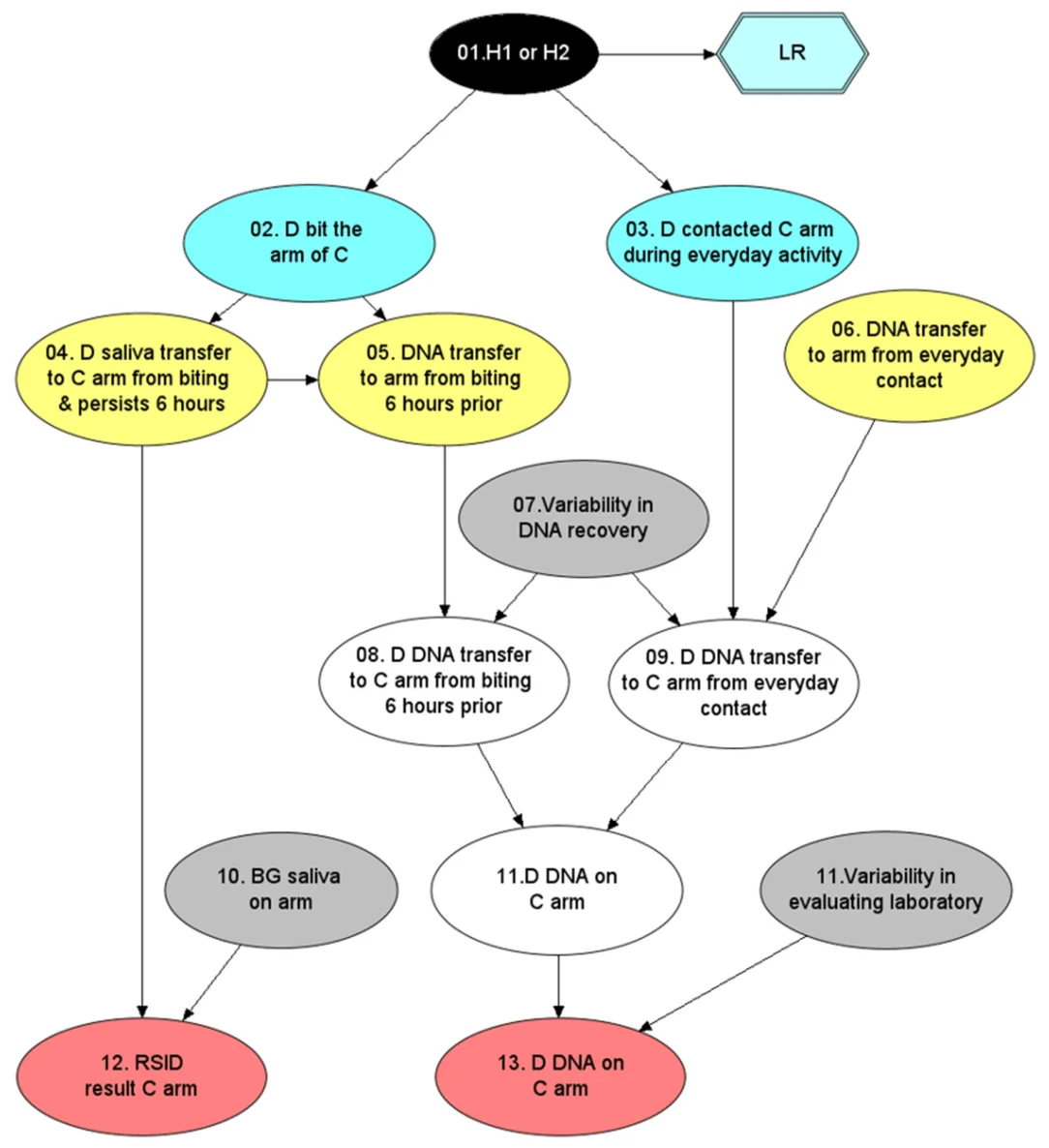
Bayesian network used to evaluate forensic observations for a case example involving allegations of biting.

There are numerous choices that can be made about the way in which an evaluation could be carried out for the given scenario. Some of these relate to the assumptions being made, some about how the data is used, and some about the modelling choices within the evaluations. We present one possible way of carrying out the evaluation that utilizes DNA amounts, and construct the evaluation framework using a tool known as a Bayesian network [35] (BN) with software HUGIN version 9.7 [36]. The BN is shown in Figure 6 and can be accessed as a Hugin file from Supplementary Material S6 (see Supplementary Material S7, sheet 1 for relevant calculations). There are a number of features within the BN shown in Figure 6 that relate to the choices made in our evaluation.

The DNA amount nodes in Figure 6 (nodes 5, 6, 8, 9, 11 and 13) have states 0 to 0, 0 to 0.01, 0.01 to 0.1, 0.1 to 1, 1 to 10, 10 to 100 and 100 to 1000 ng. The first state (0 to 0) represents a no-DNA state [37].

We include a consideration of inter-laboratory variability (demonstrated in the ReAct collaborative study [38]) using the method of Taylor et al. [39], to account for the fact that variability may exist between the laboratories carrying out the evaluative casework and the authors’ laboratories that have generated the data used in this study. To achieve this, the BN includes one variability node (node 7) applied to the DNA amounts from our work in nodes 5 and 6 to create updated distributions in nodes 8 and 9, and a second variability node applied to the DNA results in node 13 in Figure 6.

Nodes 4 and 5 relate to the amount of saliva and DNA that are transferred from a bite, which was sampled 6 h after the alleged biting. Using the results of our study, it may have been possible to model the amount of DNA transferred from a bite, and then separately consider the level of persistence expected over 6 h. We chose not to add this level of complexity to the BN, as it would result in the same LR (given that we would be using the 6 h results compared to the immediate results in order to model persistence) and there was no need to model it in this evaluation.

**Figure 7:**
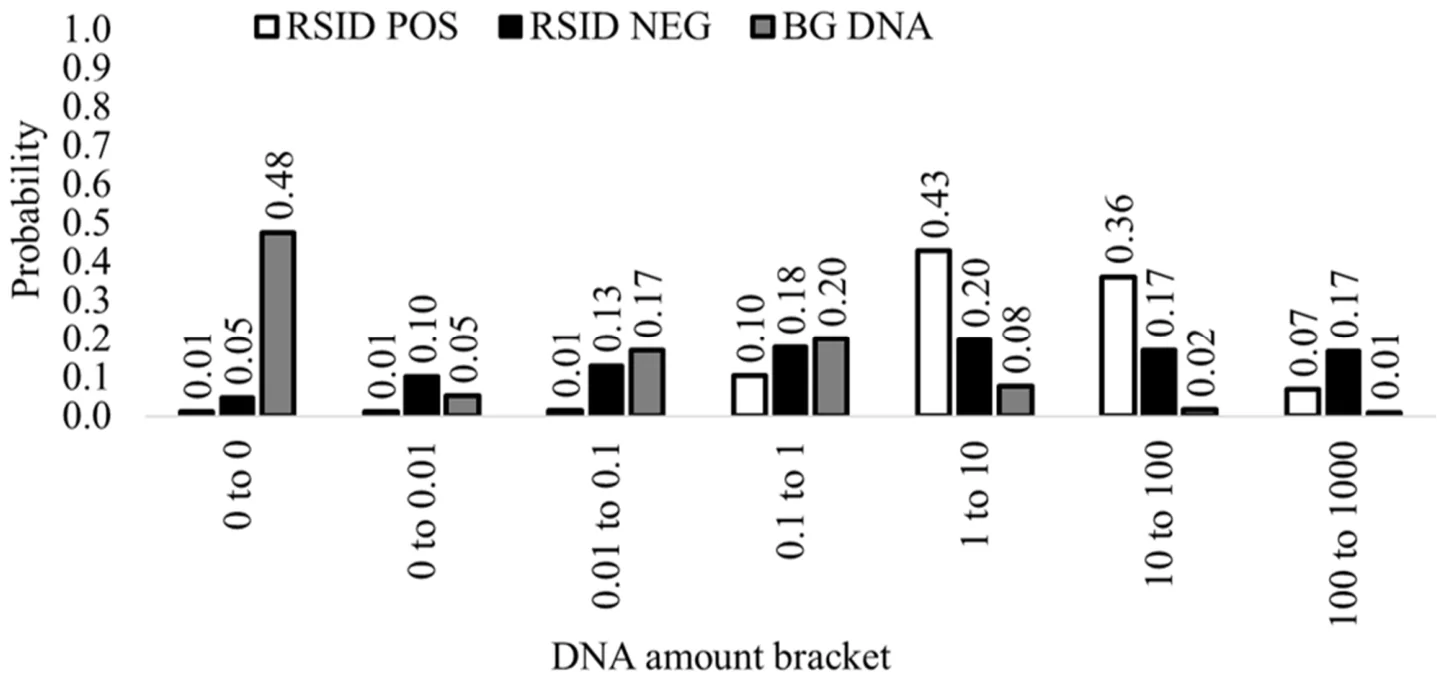
Posterior probability distribution for DNA amounts in node 5 given that saliva was transferred (white) or not transferred (black) and in node 6 for background DNA (gray).

We have included a dependency of the DNA amount on whether there was a saliva transfer to the arm from biting. To assign the probabilities presented in nodes 4 and 5, we used the data gained from the present study, whereby participants were asked to bite a section of their partner’s arm, resuming regular everyday activities until we sampled the bitten area 6 h following deposition to investigate the saliva detection rate following a period of persistence. We found that 12 of the 14 bite samples collected after 6 h generated a positive RSID-Saliva test result and therefore established a post-bite saliva detection rate. In this case, we assume the positive RSID test result was from the presence of saliva detected 6 h after the bite action, and not another substrate which could generate a false positive saliva test result (see Supplementary Material S2, sheet 2). Therefore, we consider (12 + 1)/(14 + 2) = 0.8125 as the probability for saliva transfer and (2 + 1)/(14 + 2) = 0.1875 as the probability for saliva not having transferred. We chose, then, to model the amount of DNA transferred based on whether saliva was transferred. Note that, for all DNA amount nodes, we use a Perks–Jeffreys prior on the observed counts (as explained in [40]), whereas, for the binary nodes relating to the presence or absence of saliva (nodes 4 and 10 in Figure 6), we use a uniform prior count. Figure 7 shows the posterior probability distribution of DNA amounts for the two states of saliva being present. Figure 7 shows that the two DNA distributions both have their modes in the 1 to 10 ng category; however, the DNA amount distribution relating to a negative RSID result is much flatter than the distribution for the positive RSID result. This is likely due to the lower sample number of 6 h bite samples generating a negative RSID test result (*n* = 2) upon which the distribution is created and appropriately reflects our uncertainty given this limited data.

The evaluation we carried out makes a number of assumptions. Some of these have been made to keep the example relatively simple for the purpose of demonstrating how the data obtained in this study could be used in an activity-level context. The assumptions are as follows:

There are no instances of laboratory contamination, and hence, no contamination nodes have been incorporated into the Bayesian network structure.

There are no instances of false positive or false negative RSID-Saliva test results.

The data presented in node 6 was drawn from the background samples recovered in this study, which found that all 14 background samples collected from the arms of participants generated a negative RSID-Saliva test result (see Supplementary Material S2, sheet 3).

For node 6, we rely on the data gained in this study, which indicates the amount of partner DNA detected in background samples collected from the arms of participants. From 14 background DNA samples collected from participants, 5 had their partners’ DNA detected. The posterior probability distribution for background DNA amount is shown in Figure 7 (Supplementary Material S7, sheet 2).

We also constructed the equivalent BN, as shown in Supplementary Material S8 (with conditional probability tables located in Supplementary Material S2, sheet 6), which considers the presence and absence of DNA rather than DNA amounts. We do not show the BN here but have provided it as Supplementary Material S8, and note that it is identical in architecture to the BN shown in Figure 7 of Woollacott et al. [41] (but with node naming and underlying probabilities adjusted for the current context).

**Figure 8:**
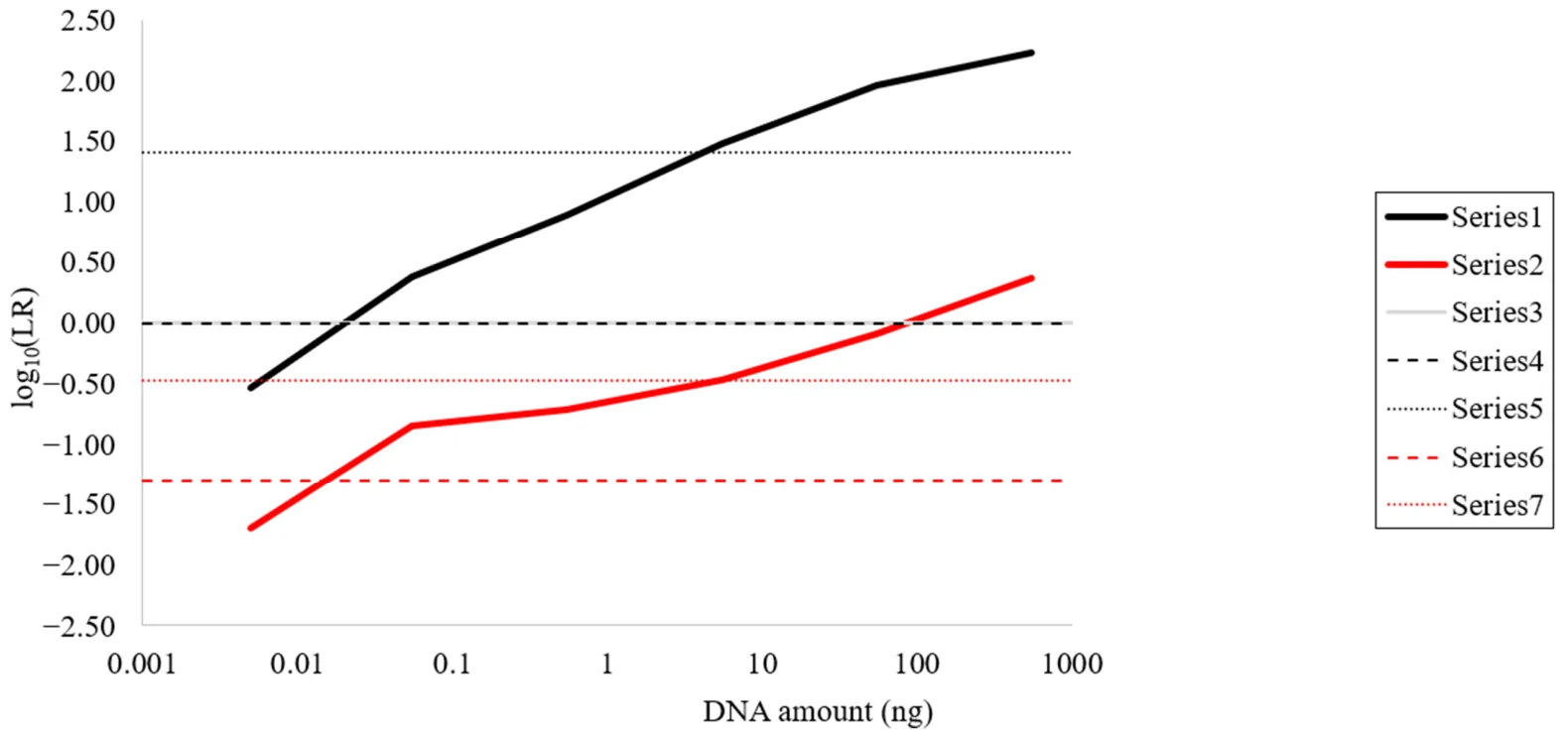
LRs obtained from evaluation of scenario given.

In Figure 8, we show the LRs that are obtained from the BN in Figure 6 and the presence or absence equivalent. We show the LRs for the full range of DNA amounts that can be obtained, and in doing so, are carrying out a sensitivity analysis of the results (as described by Taylor and Kokshoorn).

From Figure 8, it can be seen that, when considering DNA amounts, the LR ranges from exclusionary to inclusionary for either a positive or negative RSID-Saliva test result. As expected, the binary LR values fall within the range of the DNA amount LRs within an RSID test result. Of note is that, if a large amount of the defendant’s DNA was present (greater than 100 ng) and an RSID negative result was obtained, using DNA amounts would provide an LR that supports H1, whereas using the DNA would provide an LR that supports H2. This behaviour has been noted in the other literature where comparisons between binary and continuous discretization of the data are made [40]. Our example shows again the increased sensitivity to the observed results when using DNA amounts, and the greater ability to discriminate between propositions, compared to using only the presence or absence of DNA.

## 4. Discussion

This study explores the transfer and persistence of saliva and saliva-derived DNA to skin, from the actions of biting, kissing, licking and sucking compared to background and control samples taken prior to the test actions. When compared to the control and background samples, all bite, kiss, lick and suck samples recovered immediately after the test action had significantly greater amounts of total DNA. This was also the case for bite, lick and suck samples recovered 6 h post-deposition. For kiss samples, significantly more DNA was only noted when compared to background samples taken before showering. No significant difference was observed between 6 h kiss and control samples; however, 6 h kiss samples had significantly more DNA than the background samples. Both average and median amount of DNA recovered from all 6 h skin samples revealed higher quantities of total DNA detected when compared to background samples. This highlights a clear difference between the DNA amounts detected in background samples, compared to the amounts generated from different activity actions.

Comparing two sets of action samples collected at different time frames revealed that significantly more DNA was recovered immediately after each action, compared to the samples collected after 6 h, excluding bite deposits, which had no significant difference between immediate and persistence samples. These general findings align with much of the existing literature exploring the persistence of saliva on skin surfaces that reports a decline in saliva persistence between the initial deposition and up to 24 h after the transfer event [5,6].

For the deposits sampled immediately, the sucking action generated the highest median quantities of total DNA followed by lick, bite, and then kiss. For the 6 h persistence samples, the highest amounts of DNA were recovered with bite samples, followed by kiss, suck, and lick. When comparing to background samples, the kissing action resulted in an 18-fold increase in median total DNA amount when sampled immediately (7.04 ng) before a 91.5% decrease in the median amount of total DNA detected between immediate and 6 h samples (0.59 ng). When comparing background and immediate lick deposits, using the median values, we observed an approximate 38-fold increase in the total DNA detected, before it decreased by 80.29% over 6 h (2.93 ng). Between background and immediate suck samples, median amounts of total DNA increased by 231-fold before decreasing by 92.22% between immediate and 6 h suck samples. Furthermore, there was a 25-fold increase in median amounts of DNA detected on background samples, then immediately after the biting action. However, we then observed an additional 36.73% increase in the median amount of DNA detected 6 h later. This second increase may be due to subsequent DNA transfer to the area or a higher initial amount of DNA transferred to the right arms (6 h samples) compared to the left arms (immediate samples) of the participants. When comparing background and 6 h deposits, all activities still resulted in a significant increase in total DNA amounts detected after 6 h between deposition and collection. Using the median values from all sample types again, we observed a 53% increase in kiss deposits, a 7-fold increase observed in lick deposits, an 18-fold increase in suck deposits and a 59-fold increase in bite deposits. We acknowledge that, in our design, immediate samples were taken from the left arm and six-hour samples from the right arm. In principle, the left and right arms could be subjected to different activities during the six-hour interval, which may lead to different DNA persistence independent of time. Participants were asked to report any activities that might have affected the sampling areas during this period, specific to the left and right arms individually; most reported spending time at home or at the shops, activities that would not indicate systematic differences in how the left and right arms were used or exposed. Nevertheless, we cannot completely exclude arm-specific differences as a confounding factor. We also note that all activities were performed at the same anatomical locations on the arms and the action squares were not randomized across participants. As a result, it is possible that slight differences between square locations may also be confounded with the activity type. Future studies should randomize the time points, arm and anatomical location of each activity to separate time effects from arm and site effects. Additionally, our sample consisted exclusively of young, heterosexual couples, which may limit the generalizability of our findings to other age groups, relationship types, and sexual orientations; future studies should include more diverse populations to evaluate the broader applicability of these results.

For post-activity samples, 89% generated a positive saliva test result. This suggests that, across all couples, the partner DNA (at least in part) detected in these samples likely came from saliva. Notably, RSID-Saliva tests indicate the presence of amylase, not specifically its origin, and hence, these results should be interpreted with caution. Notably, the immediate deposit samples were not RSID-Saliva-tested. Although it is reasonable to assume that saliva is present immediately after conducting biting, kissing, licking and sucking actions, this was not directly verified in our study, as no saliva-specific tests were performed. Thus, caution should be exercised when inferring the biological source of the immediate samples. Similarly, because no DNA analysis was performed on the immediate samples, the total DNA could not be apportioned between the donor and the person depositing the saliva, even though it can be hypothesized that the majority of DNA immediately after the test action may belong to the partner. Notably, six samples collected 6 h post-deposition generated a negative saliva test result but revealed the presence of partner DNA, one of which was a single-source profile attributed to the partner. In the remaining samples, partners represented over 45% of DNA. Further, background and control samples also underwent RSID-Saliva testing and all generated negative results, indicating the absence of any saliva on the arms of participants before and after showering and prior to deposition. Hence, it was unlikely that any amylase generating a positive RSID test result was present on the deposit sites prior to any biting, kissing, licking, or sucking actions. All but one participant who had a negative saliva test on skin reported wearing clothing covering the areas for at least 3 h. In these instances, the partner was still representing ≥47% of the profiles, which differs from background samples, whereby partners were minor contributors, if they were detected at all. This is particularly important to understand when using this data for activity-level evaluations, as a negative saliva test result may not necessarily suggest the absence of an activity; rather, this will result in differences in profile contributions. The potential for false-positive RSID results to occur should also be considered, as this may impact how such results are interpreted in activity-level evaluations conducted. Further, the discrepancy in the saliva detection rates between pre- and post-action saliva tests can directly inform forensic scientists of the type of data expected under the different propositions, such as the ones described in Section 3.7, and are important to consider in light of the factors relevant to the body areas sampled. Factors which could result in differences in saliva detection to each area include different types of everyday activities performed, opportunities for secondary saliva transfer to occur, and environmental exposure.

For the 6 h samples, partners represented the only or majority contributor of DNA in most of these samples (83.92% or 47/56). The presence of foreign DNA not attributed to partners was rare in skin samples (14.28%, or 8/70 background and persistence skin samples), ranging from 0.4% to 43% of their respective profiles. Conversely, 55% of clothing samples detected foreign DNA from unknown sources. Notably, 8 out of 10 participants who reported wearing clothing had indicated that they wore this clothing between its last wash and its use in this study, and this could have contributed to the saliva and DNA results. It is also possible that the texture of clothing, typically consisting of woven fibres or textured fabrics, enables DNA to be retained and persist on the garment from previous wear, in contrast with how DNA persists on human skin surfaces [5,42] The findings of the present study align with prior research conducted by Kenna et al. [5], who investigated secondary transfer of saliva from skin to different fabrics. In their study, higher amounts of DNA were detected on cotton (1.22 ng) compared to leather (0.07 ng), which they attributed to the surface type, suggesting that cotton picks up and retains greater amounts of DNA than leather surfaces [5]. In this study, when comparing DNA quantities detectable on skin compared to the participants’ sleeves, the same principles can be used to understand the higher amounts of DNA that were detectable on clothing samples compared to the skin samples collected after 6 h. However, no control samples were taken from the garments worn by participants. Consequently, it remains unknown whether the partners’ DNA and saliva were already present on the clothing before the experiment, limiting the interpretive power of these samples, including possible secondary transfer from the skin of the participants.

In the samples collected at 6 h post-deposition, partner DNA was detected in all (98%) but one sample (6 h lick sample), including 16% of samples that were single-source profiles (average mixture proportions of 86%). In contrast, only 36% (5/14) of background samples generated profiles inclusionary of the donor’s partners, whereby the highest partner contribution was 72%. Profiling results highlight a difference between those generated following non-intimate activity (background samples) and post-activity samples (biting, kissing, licking or sucking), despite a 6 h delay between transfer and recovery. Ultimately, these findings highlight the value of sampling skin areas where it has been alleged that actions of biting, kissing, licking or sucking may have taken place during an assault as well as the collection of relevant clothing samples.

The presence or absence of clothing covering each deposit during the 6 h before collection could have contributed to the loss or preservation of any saliva and therefore DNA. Statistical analysis indicated that there was not a significant difference between the total and non-self amounts recovered from the arms of those who wore clothing and those who did not. Bowman et al. [43] conducted similar research reflecting on the transfer and persistence of touch DNA following mock assault scenarios whereby an “offender” was directed to grip the arms and wrists of a “victim” using different pressures. The gripped areas on the victim’s arms were sampled at 0, 3 or 24 h post-activity. The researchers found it possible for touch DNA to transfer and persist on skin, particularly with heavy pressure and friction, and further transfer to the clothing worn between initial deposition and recovery. This is also supported by Kenna et al. [5], who explored saliva persistence on legs covered by clothing, before being sampled at different time points. Their findings describe the ability to effectively recover saliva from skin up to 96 h after deposition, with persistence of saliva declining between 0 h and 24 h [5]. These findings collectively highlight the benefit of sampling clothing which may have come in contact with biological materials, transferred during an offence, aligning with the present study [43].

The BN presented in Section 3.7 exemplifies how the results in this study can be used to assign probabilities in an evaluation of activity-level propositions. In this example, we elected to use the presence and absence of saliva in combination with DNA amounts to show how this data can be used to produce a probabilistic model for evaluation of DNA results at the activity level. Figure 7 presents a series of probabilities generated from the data in this study, which can be used to generate a likelihood ratio dependent on the results instantiated. For example, if a skin sample is collected following allegations of biting, and forensic scientists have followed standard sample processing to generate a saliva positive test result with the 50 ng of the POI’s DNA present, this resulted in an LR of 92 in favour of the H1. Alternatively, if sample processing generates a negative saliva test result, but DNA processing detects 50 ng of the POI’s DNA, the LR in favour of H1 becomes 0.8. By introducing DNA amounts into this example, we have increased the discriminatory power of the model, as demonstrated by Taylor et al. [40] (this data is available in Supplementary Material S2, sheet 3). Relevant calculations used to produce this BN are shown in Supplementary Material S7, sheet 1. We also consider inter-laboratory effects, highlighting how the variability of DNA recovery across different laboratories may be incorporated into the evaluation [38]. The probabilities presented in this example were generated from DNA profiling results from one study, which we acknowledge as a limitation. We also acknowledge that differences in extraction temperature, lysis buffer volume and cycle number may vary between laboratories (including laboratory 1 and 2 used in this research, which are outlined in Supplementary Material S5), presenting a limitation. Therefore, if future studies wish to incorporate our results in their evaluations, potential differences in recovery methods should be considered [38]. The collection of further samples, such as control samples from skin areas adjacent to each square containing an immediate or persistent bite, kiss, lick or suck deposit, and the collection of background and control samples from clothing, are recognized as possible improvements in the present study. These improvements to the experimental design would help ascertain if any of the deposited saliva had transferred to surrounding skin areas during the 6 h period before recovery. Similarly, a control sample collected from the clothing prior to being worn during the 6 h before sampling may have allowed for a stronger understanding of whether the DNA recovered on clothing samples was a result of transfer from the saliva deposits, or if this DNA was already present prior to experiment commencement.

## 5. Conclusions

Throughout this research, the transfer and 6 h persistence of saliva on skin surfaces, from actions of biting, kissing, licking and sucking, was explored, generating data useful to various casework circumstances. The DNA quantities and resultant profiles before an activity (background and control samples) and after an activity (both immediately and after a 6 h period of persistence) were assessed and compared during this research. There was a significant initial increase in total amounts of DNA detectable on skin following all four actions. However, a decrease over the 6 h between transfer and recovery was then observed across all samples, excluding the biting deposits, which saw an additional increase in DNA. Saliva from all persistent skin samples generated a 6 h detection rate of 86% for bite, 71% for kiss and 100% for lick and suck actions and a 60% 6 h detection rate on relevant clothing areas, as indicated from RSID-Saliva test results. Partners were detected in 98% of 6 h persistence samples, with partner mixture proportions representing significantly greater proportions in all persistence samples when compared to background samples. The overall findings of this research highlight the value of collecting skin and clothing samples with suspected transfer of salivary DNA, providing important DNA evidence valuable to informing forensic casework.

## Data Availability

All data and original contributions presented in this research are included throughout the manuscript and in the Supplementary Material files provided. Further inquiries can be made by contacting the corresponding author listed.

## Supplementary Materials

The following supporting information can be downloaded at https://www.mdpi.com/article/10.3390/genes17101229/s1, File S1: Questionnaire; File S2: Raw data; File S3: statistical test results; File S4: R Studio script; File S5: Project Methodology; File S6: Bayesian network 1; File S7: Bayesian network 1 calculations; File S8: Bayesian network 2.

## Abbreviations

The following abbreviations are used in this manuscript:

DNA-TPPR	DNA Transfer, prevalence, persistence and recovery
STR	Short Tandem Repeat
RSID	Rapid Stain Identification
LR	Likelihood Ratio
BN	Bayesian Network
NoC	Number of Contributors
RFU	Relative Fluorescent Unit
