# Supplementary material for: A Comparison of Saliva and DNA Transfer and Persistence on Skin Immediately and 6 h Following Biting, Kissing, Licking and Sucking
Source: Genes (Basel). 2026 Oct 3;17(10):1229. doi: 10.3390/genes17101229 (PMC13704104; doi:10.3390/genes17101229)
Supplement: Supplementary file 1 [file genes-17-01229-s001.zip › Supplementary material 1.pdf]

Flinders University

Participant Code: ____________

PARTICIPANT QUESTIONNAIRE

Project Title: Persistence of saliva on human skin and its implications on forensic DNA results interpretation

Please tick/fill in your answers.

*Please note that **you can skip** questions you don't wish to answer.*

Section S1: General Questions

1. **Sex**

☐ Male
☐ Female
☐ Other
☐ Prefer not to specify

2. **Age**

☐ 18-25
☐ 26-35
☐ 36-45
☐ 46-55
☐ 56-65
☐ 66-75
☐ 76-85
☐ 86-95
☐ Prefer not to specify

3. **Which hand is your dominant hand?**

☐ Left
☐ Right
☐ Ambidextrous

The questions 4 to 12 will assist scientists in the understanding of the factors that may influence the detection of DNA on your arms. Skin conditions, use of moisturisers and arm hairiness can result in lower DNA amounts detected and inhibit some of the DNA processes while facial touching is known to load hands with DNA from the face that may contain traces of saliva.

4. **Do you have any skin conditions (yes/no)? If yes, please list conditions below:**

☐ Yes
☐ No
☐ Prefer not to specify

____

____

____

Flinders University

Participant Code: ____________

5. Do you bite your nails?

☐ Yes
☐ No
☐ Prefer not to specify

6. Do you use moisturiser for your hands?

☐ Yes (if yes, specify which part of your hands, what you used to moisturise your hands and when you last moisturised them in the lines below)
☐ No

____

____

7. Do you use moisturiser for your arms and shoulder?

☐ Yes (if yes, specify which part of your arms, and when the last time your arms were moisturised in the lines below)
☐ No

____

____

8. Do you use sanitizer/disinfectant for your hands?

☐ Yes (if yes, specify which part of your hands, the type of disinfectant/sanitizer you use and the last time you used sanitizer on your hands in the lines below)
☐ No

____

____

9. Do you use sanitizer/disinfectant on your arms?

☐ Yes (if yes, specify which part of your arms, and the last time you used sanitizer and the type you used on your arms in the lines below)
☐ No

____

____

Participant Code: ____________

10. How frequently do you touch your face per hour (including your neck, ears and/or hair)? Please provide your best estimate.

☐ 0 times
☐ 1-5 times
☐ 6-10 times
☐ 11-20 times
☐ 20-30 times
☐ > 30 times
☐ Prefer not to specify

11. How frequently do you touch your left arm with your right hand per hour? Please specify which area(s) you have recently contacted below.

☐ 0 times
☐ 1-5 times
☐ 6-10 times
☐ 11-20 times
☐ 20-30 times
☐ > 30 times
☐ Prefer not to specify

____

____

____

12. How frequently do you touch your right arm with your left hand per hour? Please specify which area(s) you have recently contacted below.

☐ 0 times
☐ 1-5 times
☐ 6-10 times
☐ 11-20 times
☐ 20-30 times
☐ > 30 times
☐ prefer not to specify

____

____

____

Flinders University

Participant Code: ____________

The questions 13 to 18 help scientists understand if the foreign DNA present on the arms may have been inadvertently transferred from common activities such as sharing a towel or a bed.

13. What are your living arrangements?

- ☐ Live alone
- ☐ Live with flatmate(s)
- ☐ Live with partner
- ☐ Live with partner and children
- ☐ Live with partner and flatmate (s)
- ☐ Live with parents and/or siblings
- ☐ Other (please provide details below)
- ☐ Prefer not to specify

____

____

14. If you live with at least one other individual, please provide the number of total co-habitants below

____

____

15. Do you share a bed?

- ☐ Yes
- ☐ No
- ☐ prefer not to say

16. Do you share a primary bathroom?

- ☐ Yes
- ☐ No
- ☐ prefer not to say

17. Do you share a towel to dry your arms?

- ☐ Yes
- ☐ No
- ☐ prefer not to say

18. Do you share a towel to dry your hands?

- ☐ Yes
- ☐ No
- ☐ Prefer not to say

Flinders University

Participant Code: ____________

Section S2: Background samples (prior to taking a shower).

The questions in section two will help the researchers to better understand the results (both saliva and DNA) obtained from the background (i.e. prior to the experimental actions) samples. It is known that biological material can inadvertently transfer to the body surfaces from normal everyday activities such as intimate activities. It is also known that some activities, such as washing or wearing clothes, can result in lower-than-expected detection of DNA. Hence, understanding what happened prior to sample collection will assist in results interpretation.

19. How many hours ago did you last wash yourself (prior to taking background samples and showering)?

____

____

20. How did you last wash yourself?

☐ Bath

☐ Shower

☐ other (eg. went for a swim without showering)

☐ prefer not to say

If other, please specify below.

____

____

21. When was the last time that you can recall each section of your arms were touched by anyone other than yourself (please provide your best estimate)?

1	2	3	4	5	6
☐ 0-2 hours ago<br>☐ 2- 8 hours ago<br>☐ 8-16 hours ago<br>☐ > 24 hours ago<br>☐ > 36 hours ago<br>☐ > 1 week ago	☐ 0-2 hours ago<br>☐ 2- 8 hours ago<br>☐ 8-16 hours ago<br>☐ > 24 hours ago<br>☐ > 36 hours ago<br>☐ > 1 week ago	☐ 0-2 hours ago<br>☐ 2- 8 hours ago<br>☐ 8-16 hours ago<br>☐ > 24 hours ago<br>☐ > 36 hours ago<br>☐ > 1 week ago	☐ 0-2 hours ago<br>☐ 2- 8 hours ago<br>☐ 8-16 hours ago<br>☐ > 24 hours ago<br>☐ > 36 hours ago<br>☐ > 1 week ago	☐ 0-2 hours ago<br>☐ 2- 8 hours ago<br>☐ 8-16 hours ago<br>☐ > 24 hours ago<br>☐ > 36 hours ago<br>☐ > 1 week ago	☐ 0-2 hours ago<br>☐ 2- 8 hours ago<br>☐ 8-16 hours ago<br>☐ > 24 hours ago<br>☐ > 36 hours ago<br>☐ > 1 week ago

☐ Prefer not to say

22. Was the last person who touched your arms your partner who is also participating in this study?

☐ Yes

☐ No

☐ Prefer not to say

Participant Code: ____________

23. Have you been wearing clothing covering your left arm prior to sampling?

☐ Yes (please answer question a. below)

☐ No (please answer question b. below)

☐ Prefer not to say

24. If you answered yes to question 23 above, please indicate which area had clothing contacting it, and for how long

1	2	3	4	5	6
☐ 0-1 hours<br>☐ 1-2 hours<br>☐ 2- 4 hours<br>☐ 4-8 hours<br>☐8-12 hours<br>☐ 12-24 hours<br>☐ > 24 hours	☐ 0-1 hours<br>☐ 1-2 hours<br>☐ 2- 4 hours<br>☐ 4-8 hours<br>☐8-12 hours<br>☐ 12-24 hours<br>☐ > 24 hours	☐ 0-1 hours<br>☐ 1-2 hours<br>☐ 2- 4 hours<br>☐ 4-8 hours<br>☐8-12 hours<br>☐ 12-24 hours<br>☐ > 24 hours	☐ 0-1 hours<br>☐ 1-2 hours<br>☐ 2- 4 hours<br>☐ 4-8 hours<br>☐8-12 hours<br>☐ 12-24 hours<br>☐ > 24 hours	☐ 0-1 hours<br>☐ 1-2 hours<br>☐ 2- 4 hours<br>☐ 4-8 hours<br>☐8-12 hours<br>☐ 12-24 hours<br>☐ > 24 hours	☐ 0-1 hours<br>☐ 1-2 hours<br>☐ 2- 4 hours<br>☐ 4-8 hours<br>☐8-12 hours<br>☐ 12-24 hours<br>☐ > 24 hours

25. If you answered no to question 23 above, when was the last time you had clothing contact each area on your arm?

1	2	3	4	5	6
☐ 0-2 hours ago<br>☐ 2- 8 hours ago<br>☐ 8-16 hours ago<br>☐ > 24 hours ago<br>☐ > 36 hours ago<br>☐ > 1 week ago	☐ 0-2 hours ago<br>☐ 2- 8 hours ago<br>☐ 8-16 hours ago<br>☐ > 24 hours ago<br>☐ > 36 hours ago<br>☐ > 1 week ago	☐ 0-2 hours ago<br>☐ 2- 8 hours ago<br>☐ 8-16 hours ago<br>☐ > 24 hours ago<br>☐ > 36 hours ago<br>☐ > 1 week ago	☐ 0-2 hours ago<br>☐ 2- 8 hours ago<br>☐ 8-16 hours ago<br>☐ > 24 hours ago<br>☐ > 36 hours ago<br>☐ > 1 week ago	☐ 0-2 hours ago<br>☐ 2- 8 hours ago<br>☐ 8-16 hours ago<br>☐ > 24 hours ago<br>☐ > 36 hours ago<br>☐ > 1 week ago	☐ 0-2 hours ago<br>☐ 2- 8 hours ago<br>☐ 8-16 hours ago<br>☐ > 24 hours ago<br>☐ > 36 hours ago<br>☐ > 1 week ago

26. Please specify which section of your arms were covered when you last wore clothing

1	2	3	4	5	6
☐ covered<br>☐ uncovered	☐ covered<br>☐ uncovered	☐ covered<br>☐ uncovered	☐ covered<br>☐ uncovered	☐ covered<br>☐ uncovered	☐ covered<br>☐ uncovered

27. Has another individual worn the upper clothes that last contacted your arms since they were last washed?

☐ Yes

☐ No

☐ Prefer not to specify

Participant Code: ____________

28. If yes, was this individual your partner in this experiment?

☐ Yes
☐ No
☐ Prefer not to specify

29. Time since last sexual activity?

☐ 0-2 hours
☐ 2- 8 hours
☐ 8-16 hours
☐ > 24 hours
☐ > 36 hours
☐ > 1 week
☐ Prefer not to say

30. During your last sexual activity were your arm(s) kissed, bitten or licked by your partner?

☐ Yes (Please, specify which arm(s) and which parts of your arm(s) in the lines below)
☐ No
☐ Prefer not to specify

____

____

____

31. When was the last time you made contact to your mouth (either outside or inside of your mouth) with your hands for any other reason? (e.g. Smoking, vaping, taking chewing gum out, eating, applying lip balm etc.) Please specify what action/activity resulted in you contacting your mouth below.

☐ < 30 minutes
☐ 30 minutes to 1 hour
☐ 1-3 hours
☐ > 3 hours

____

____

____

Flinders University

Participant Code: ____________

32. When did you wash your hands last?

☐ < 30 minutes

☐ 30 minutes to 1 hour

☐ 1-3 hours

☐ > 3 hours

33. When was the last time you used the bathroom?

☐ < 30 minutes

☐ 30 minutes to 1 hour

☐ 1-3 hours

☐ > 3 hours

34. What type of bathroom was this?

☐ Private (at home)

☐ At home of friend or family

☐ Public

☐ Other (Please specify)

____

____

35. Where have you spent most, and second most, of your time today prior to taking background samples?

Most: Duration: ____________

☐ Work

☐ Home

☐ In your car

☐ Shopping

☐ Non-communal spaces

☐ Communal spaces

☐ Other (Please specify below)

____

____

____

Second most: Duration: ___________

- ☐ Work
- ☐ Home
- ☐ In your car
- ☐ Shopping
- ☐ Non-communal spaces
- ☐ Communal spaces
- ☐ Other (please specify below)

____

____

____

36. Are you aware of any major activities/factors that may impact the presence of DNA from surfaces collected? (e.g. Went for a run and sweated a lot, used a dirty towel, went to the shops and had my arms exposed etc.)

- ☐ Yes
- ☐ No
- ☐ Prefer not to specify

If yes, provide details if you wish (optional)

____

____

____

____

____

____

____

____

____

____

Flinders University

Participant Code: ____________

The questions in section S3 will assist the scientists in interpreting the results from samples obtained post experimental activity. Everything that you do post activity can influence the types of the results obtained. Hence these questions will assist in results interpretation in light of these activities.

Section S3: 6-hour experimental samples (post biting/licking activity) – relating to right arm.

37. Please indicate which sections on your arm (squares 1-6) were covered by clothing during the 6-hours post biting/licking activity and for how long on the timelines below. Please also indicate the material on the line below for each section (e.g. Denim sleeve).

a. ____

Section 1

0h 0.5h **1h** 1.5h **2h** 2.5h **3h** 3.5h **4h** 4.5h **5h** 5.5h **6h**

b. ____

Section 2

0h 0.5h **1h** 1.5h **2h** 2.5h **3h** 3.5h **4h** 4.5h **5h** 5.5h **6h**

c. ____

Section 3

0h 0.5h **1h** 1.5h **2h** 2.5h **3h** 3.5h **4h** 4.5h **5h** 5.5h **6h**

d. ____

Section 4

0h 0.5h **1h** 1.5h **2h** 2.5h **3h** 3.5h **4h** 4.5h **5h** 5.5h **6h**

e. ____

Section 5

0h 0.5h **1h** 1.5h **2h** 2.5h **3h** 3.5h **4h** 4.5h **5h** 5.5h **6h**

f. ____

Section 6

0h 0.5h **1h** 1.5h **2h** 2.5h **3h** 3.5h **4h** 4.5h **5h** 5.5h **6h**

Participant Code: ____________

If necessary, please provide further details if multiple upper garments were worn during the period since showering and sampling, only reporting on garments which made direct contact with the arm during this period. Please provide a brief description of the clothing and roughly how long it was worn for (e.g. Cotton long sleeve shirt for 2 hours):

____

____

____

____

38. What type of upper clothing have you been wearing post biting/licking activity?

☐ No sleeves

☐ Short sleeves

☐ ¾ sleeves

☐ Full sleeves

39. Have you previously worn the upper clothes you wore during the 6-hour period post biting/licking activity since it was last washed?

☐ Yes (Please specify below approximately how many times and total number of hours)

☐ No

☐ prefer not to specify

____

____

40. Have you shared the upper clothes that you were wearing during the 6-hour post biting/licking activity (i.e. allowed someone else to wear it) since they were last washed?

☐ Yes

☐ No

☐ Prefer not to specify

41. If yes, was this individual your partner in this experiment?

☐ Yes

☐ No

☐ Prefer not to specify

Participant Code: ____________

42. How often did you touch your face in the 6- hour period post biting/ licking activity? See options from background section

☐ 0 times
☐ 1-5 times
☐ 6-10 times
☐ 11-20 times
☐ 20-30 times
☐ > 30 times
☐ prefer not to specify

43. How many times did you contact your mouth (either outside or inside of your mouth) with your hands for any other reason during the 6-hour period post biting/licking activity?

____

44. Please specify what action/activity(s) resulted in you contacting your mouth below? (e.g. Smoking, vaping, taking chewing gum out, eating, applying lip balm etc.). Select all that apply.

☐ Eating/drinking
☐ Smoking/vaping
☐ Taking out chewing gum
☐ Biting nails
☐ Applying lip balm/lip gloss/lipstick
☐ Other (please specify below)
☐ Prefer not to specify

____

____

____

45. How frequently did you touch your other areas of your face (including your neck, ears and/or hair) per hour during the 6-hours post biting/licking activity (please provide your best estimate)?

☐ 1-10 times
☐ 11-20 times
☐ 20-30 times
☐ > 30 times
☐Prefer not to specify

Flinders University

Participant Code: ____________

46. How frequently did you touch your right arm during the 6-hours post biting/licking? Please specify which area(s) you have contacted below.

1	2	3	4	5	6
☐ 0 times<br>☐ 1-2 times<br>☐ 3-5 times<br>☐ 6-10 times<br>☐ >10 times	☐ 0 times<br>☐ 1-2 times<br>☐ 3-5 times<br>☐ 6-10 times<br>☐ >10 times	☐ 0 times<br>☐ 1-2 times<br>☐ 3-5 times<br>☐ 6-10 times<br>☐ >10 times	☐ 0 times<br>☐ 1-2 times<br>☐ 3-5 times<br>☐ 6-10 times<br>☐ >10 times	☐ 0 times<br>☐ 1-2 times<br>☐ 3-5 times<br>☐ 6-10 times<br>☐ >10 times	☐ 0 times<br>☐ 1-2 times<br>☐ 3-5 times<br>☐ 6-10 times<br>☐ >10 times

☐ Prefer not to specify

47. What kind of contact did you make to each section of your right arm during the 6-hours post biting/licking activity? Please tick all that apply

1	2	3	4	5	6
☐ gripping<br>☐ scratching<br>☐ rubbing<br>☐ other, please specify below	☐ gripping<br>☐ scratching<br>☐ rubbing<br>☐ other, please specify below	☐ gripping<br>☐ scratching<br>☐ rubbing<br>☐ other, please specify below	☐ gripping<br>☐ scratching<br>☐ rubbing<br>☐ other, please specify below	☐ gripping<br>☐ scratching<br>☐ rubbing<br>☐ other, please specify below	☐ gripping<br>☐ scratching<br>☐ rubbing<br>☐ other, please specify below

☐ Prefer not to specify

____

____

42. Where have you spent most, and second most, of your time today post biting/licking activity?

Most: Duration: ____________

- ☐ Work
- ☐ Home
- ☐ In your car
- ☐ Shopping
- ☐ Non-communal spaces
- ☐ Communal spaces
- ☐ Other (please specify below)

____

____

____

Participant Code: ____________

43. Second most: Duration: ____________

- ☐ Work
- ☐ Home
- ☐ In your car
- ☐ Shopping
- ☐ Non-communal spaces
- ☐ Communal spaces
- ☐ Other (please specify below)

44. Please list/describe the activities you performed in the 6-hours post biting/licking activity which may impact the persistence of saliva on your arm (e.g. Sat on the couch watching TV with a blanket contacting your arm for 1 hour, etc)

45. Are you aware of any major activities/factors that may impact the presence of DNA from surfaces collected? (e.g. went for a run and sweated a lot)

- ☐ Yes
- ☐ No

If yes, provide details if you wish (optional)
